# Whole Body Vibration Training is Osteogenic at the Spine in College-Age Men and Women

**DOI:** 10.2478/v10078-012-0006-8

**Published:** 2012-04-03

**Authors:** Gianna C. Ligouri, Todd C. Shoepe, Hawley C. Almstedt

**Affiliations:** 1 Human Performance Laboratory, Department of Health and Human Sciences, Loyola Marymount University, Los Angeles, USA.

**Keywords:** osteoporosis, peak bone mass, bone mineral density, whole body vibration, resistance training

## Abstract

Osteoporosis is a chronic skeletal disease characterized by low bone mass which is currently challenging the American health care system. Maximizing peak bone mass early in life is a cost-effective method for preventing osteoporosis. Whole body vibration (WBV) is a novel exercise method with the potential to increase bone mass, therefore optimizing peak bone and decreasing the risk for osteoporotic fracture. The aim of this investigation was to evaluate changes in bone mineral density at the hip, spine, and whole body in college-age men and women who underwent a WBV training protocol. Active men (n=6) and women (n=4), ages 18–22 participated in the WBV training; while an additional 14 volunteers (1 male, 13 female) served as controls. All participants completed baseline and follow-up questionnaires to assess health history, physical activity, dietary intake, and menstrual history. The WBV training program, using a Vibraflex 550, incorporated squats, stiff-leg dead lifts, stationary lunges, push-up holds, bent-over rows, and jumps performed on the platform, and occurred 3 times a week, for 12 weeks. Dual energy x-ray absorptiometry (Hologic Explorer, Waltham, MA, USA) was used to assess bone mineral density (BMD, g/cm^2^). A two-tailed, t-test identified significantly different changes in BMD between the WBV and control groups at the lateral spine (average change of 0.022 vs. −0.015 g/cm^2^). The WBV group experienced a 2.7% and 1.0% increase in BMD in the lateral spine and posterior-anterior spine while the control group decreased 1.9% and 0.9%, respectively. Results indicate that 12 weeks of WBV training was osteogenic at the spine in college-age men and women.

## Introduction

Osteoporosis is a skeletal disease characterized by low bone mass, resulting in an increased risk for fracture. This disease is a major public health concern contributing annually to an estimated 2 million fractures costing $17 billion to the American health care system (Burge et al., 2007). Obtaining an optimal peak bone mass (PBM), which is the highest potential bone mineral density (BMD) achieved during young adult life, is vital for preventing osteoporosis. Peak bone mass is attained through skeletal maturation and thus occurs in the third decade of life (Recker et al., 1992). If an individual does not reach optimal PBM, they are at greater risk for osteoporotic-induced fracture. However, research shows that a 3–5% increase in bone mass retained though adulthood may decrease future fracture risk by 20–30% (Wasnich and Miller, 2000). Because lifestyle factors such as diet and exercise have been shown to influence bone mass values by up to 40%, it is important to explore various exercise modalities to determine effective methods of attaining optimal PBM (Pocock et al., 1987). Healthful dietary intake, mechanical loading, including performing resistance training, have shown to be effective at increasing BMD in young adults and thereby lowering risk of osteoporosis (Kohrt et al., 2004).

Whole body vibration (WBV) is a newly recognized training modality with the potential to increase strength, reverse sarcopenia, and improve bone mineral density (Niewiadomski et al., 2005; Rittweger, 2010). Whole body vibration utilizes the physical mechanics of energy transfer through vibration to provoke muscle contractions when standing on the active platform. Cycles of muscle contraction and relaxation correspond to the frequency of the vibrating platform (Rittweger, 2010). While there are various types of energy transfer provided by different types of WBV platforms, the platform utilized in this study operates using a side-alternating or “teeter-totter” fashion (Rittweger, 2010). This method employs sinusoidal oscillations, which cause the platform to alternate over a center fulcrum in a steady wave-like motion, sending consistent vibrations through the musculature.

Recent experimental research has suggested WBV can improve strength of both muscle and bone (Niewiadomski et al., 2005; Verschueren et al., 2004). Jacobs and Burns observed an acute increase in flexibility and torque in lower-extremity musculature of male and female participants who stood on a WBV platform for six minutes (Jacobs and Burns, 2009). Hong and colleagues reported an increase in muscular performance at the shoulder joint after acute exposure to vibration via hand placement while holding a push-up position on the platform (Hong et al., 2010). Other muscular benefits of WBV were observed by McBride et al. (2010), who reported a short-term increase in muscle force output following a whole body vibration and static squat program. Improvements in bone health with WBV training were observed by Gilsanz et al. (2006) who reported an increase in trabecular bone mineral density of the spine and cortical bone area of the femur in young women with a history of fracture and low BMD. These results were observed after the participants underwent a 12-month WBV protocol that consisted of standing on the platform vibrating at 30 Hz for 10 minutes daily. A similar study by Beck et al. (2006) reported a 2% increase in BMD at the non-dominant hip in premenopausal women completing a WBV program requiring 10 minutes of vibration exposure, 2 times per day for 12 months. These findings suggest that WBV is effective when the participants stand static on the platform. However, little research has been done on the osteogenic benefits of WBV in conjunction with dynamic exercises. Because weight-bearing exercise improves BMD in a site-specific manner, it is important to consider a specific, dynamic program that targets the bone tissue at clinically relevant sites such as the hip and spine. Previous work has shown squat and deadlift exercises to be osteogenic specifically at the spine (Almstedt et al., 2011). Therefore, the purpose of this investigation was to evaluate the osteogenic potential of WBV training with dynamic exercise at improving bone health in young individuals, thereby optimizing peak bone mass development and lowering future risk for osteoporosis.

## Methods

Active college-age men and women were recruited for a bone study in September and October of the fall semester. Volunteers from this group were invited to participate in a 12-week WBV training program designed to improve BMD or serve as controls. WBV training began in January and continued for 12 weeks with following up testing taking place in the first week of May. Baseline testing occurred in September/October and follow-up testing took place in May immediately after 12 weeks of WBV training. There was an average of 29 weeks between assessments, with the intervention taking place in the final 12 weeks. The exercise intervention consisted of sessions lasting 20–30 minutes which were performed at a vibration frequency range of 15–26 Hz, 3 days per week. Both WBV participants and controls completed questionnaires to assess physical activity, calcium intake, and menstrual history. Dual energy x-ray absorptiometry (DXA) was used to measure the dependent variable, BMD (g/cm^2^) of the hip, spine, and whole body. Change in BMD between the independent variable assignment of WBV training or no training (controls) was analyzed using a t-test.

### Participants

Twenty-five active, college-age men (n=7) and women (n=18) volunteered to participate in a whole body vibration training program or serve as controls. Of these, 14 were control participants (1 male, 13 females) who were asked to continue their normal diet and exercise patterns throughout the period of study. The remaining 11 participants (6 males, 5 females) underwent a 12-week whole body vibration training program.

All participants were between the ages of 18 and 22 and had similar activity levels and body mass index. Volunteers had no current musculoskeletal injuries and no prior exposure to WBV. Unfortunately, one female WBV participant dropped out of the training program due to a lower leg injury, which occurred outside the training program, therefore her data is not included in the analysis. Table 1 shows the means and standard deviations for the demographic characteristics of the participants at baseline.

The WBV and control groups were similar in age, BMI, calcium intake, and physical activity; however, the WBV participants had a significantly lower percent body fat and a greater lean body mass. Before commencing the training program, all WBV volunteers and controls were informed about the potential risks of participation and written approval was obtained from participants. The protocol explained here was approved by the Loyola Marymount University Institutional Review Board for Human Subjects Research.

### Procedures

Body height in cm was determined by use of a stadiometer (Seca Accu-Hite, Columbia, MD) and body mass was measured in kg on an electronic scale (Tanita BWB-627A, Tokyo, Japan). BMI was then calculated using this data by dividing body mass in kg by the square of the height in meters. Self-administered questionnaires were completed by all participants under the supervision of a research assistant to determine multiple variables such as physical activity, calcium intake, and menstrual function. Questionnaires were administered before beginning the WBV training program to determine baseline data. Testing was repeated again after completion of the 12-week training program. The Aerobic Center Longitudinal Study Physical Activity Questionnaire was used to assess participants’ intensity and duration of exercise in metabolic equivalents (MET-hours per week) by using age, body mass, hours per week of physical activity, and intensity of activity. This questionnaire has previously been validated with this population (Pereira et al., 1997). The Block 2005 Food Frequency Questionnaire (NutritionQuest, Berkeley, CA), which evaluates food and nutrient intake in the previous year, was used to assess participants’ baseline calcium intake. At the end of the study period, participants completed the LMU Rapid Assessment Method Questionnaire (LMU RAM) designed to evaluate daily calcium intake for the past 7 days. Both methods of calcium assessment are valid and have been deemed reliable for this population (Block et al., 1990; Henry and Almstedt, 2009). All female participants described their menstrual history for the previous 3 months and reported any use of hormonal contraceptives in the menstrual history questionnaire.

### Whole Body Vibration Training Program

**Figure 1:**
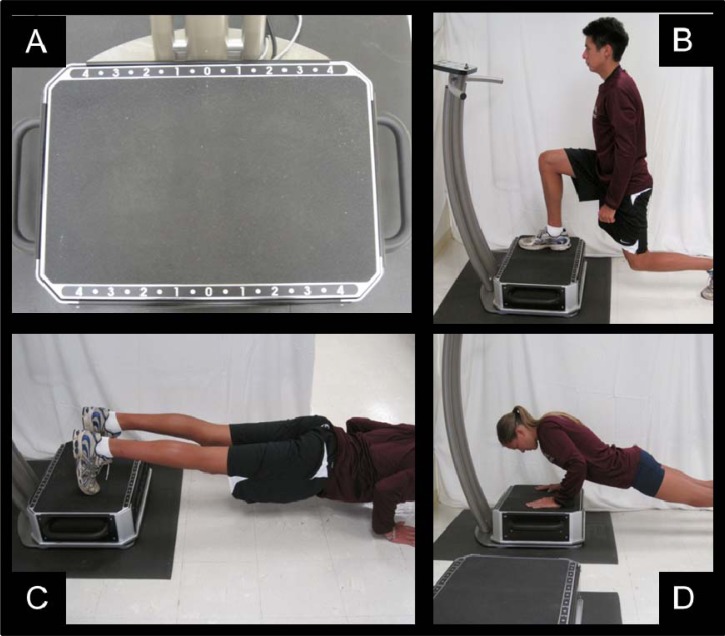
Whole Body Vibration Platform and Exercises A. Whole body vibration platform, participants were instructed to place their feet at position 2 indicated at the top and bottom of the platform B. Lunging exercise on the whole body vibration platform C. *Push-up hold completed with feet placed on whole body vibration platform* D. *Push-up hold completed with hands placed on whole body vibration platform*

The optimal amplitude, frequency, and duration of vibration exposure for bone health are yet to be clearly defined (Totsy de Zepetnek et al., 2009). The training program was designed with the objective of increasing BMD and therefore weight-bearing exercises were selected specifically with this goal in mind. Training was performed on a Vibraflex 550 (also called the Galileo 2000, Novotec, Pforzheim, Germany) which utilizes a side-to-side displacement mimicking a “teeter-totter” motion based around a center fulcrum (Figure 1). The parameters selected here were based on previous research involving a similar WBV platform which was found to be effective for eliciting a muscular, hormonal, or bone response (Ambrecht et al., 2010; Cardinale and Bosco, 2003; Gusi et al., 2006; Iwamoto et al., 2005; Russo et al., 2003). The progressive overload principal was incorporated, creating a nonlinear periodized program that provided for fluctuations in volume, intensity, and exercise complexity over the training period. WBV participants completed 12 weeks of training, 3 times per week under the close supervision of trained research assistants. Each workout session lasted 20–30 minutes and included one minute rest period between each exercise set. Before beginning the WBV protocol, a safety orientation was held to familiarize the participants with the correct exercise form. In order to minimize the displacement of the head during exercise, participants were instructed to avoid locked-out joint positions at all times by maintaining slight (∼5°) flexion of the ankles, knees, elbows, and hips during static set (e.g. standing) and through the concentric-to-eccentric transition of each dynamic repetition. In the first two weeks, some exercises were performed on the floor without vibration in order to develop mastery of technique and therefore reduce the risk of injury, however only those completed on the platform are accounted for in Table 2. When volunteers could demonstrate proper exercise mechanics on the floor, they progressed to the platform. All participants had performed the entire protocol on the platform by the sixth workout session (i.e. second week). Volume undulated through the duration of the program where the total number of sets varied from 4–11 and total time of vibration per session varied from 165–540 seconds (2.7–9 min). The protocol consisted of a combination of squats, stiff-leg deadlifts, stationary lunges, push-up holds, bent-over rows, and jumps onto and off of the platform. Table 2 shows a detailed account of the WBV program with time in seconds, number of sets, and frequency in Hz (number of vibration cycles per second). Participants were asked to place their feet or hands at position 2, marked on the platform (Figure 1A), where they experienced a peak-to-peak displacement of 4.16 mm therefore an amplitude of 2.08 mm.

In general, workout 1 of each week incorporated squats, stiff-leg dead lifts, push-up holds, and jumps. Workout 2 of each week included stiff-leg dead lift, stationary lunges, bent-over rows, and jumps. Workout 3 was a combination of all exercises from the previous two workouts, but with reduced sets in order to maintain the duration of the workout. Every workout began with a warm-up of standing on the platform with slight flexion throughout the lower extremity as described previously. Initially, WBV squats were performed in a statically held position at parallel thigh depth so that the participants could become familiar with the form. WBV squats on the platform were performed initially with hands fully gripping the supporting handlebars. By week 4, all exercisers had progressed to dynamically squatting the full range of motion without the aid of the handles. In week 6, heel and toe raises were introduced into the squat protocol with smooth transitions mandated between each segment. For example, a single repetition occurred in a cyclical fashion where a squat concluded near full extension, and was followed immediately by a full repetition of the heel raise exercise, which was then followed by a toe raise prior to initiating the next squat repetition. In week 11, resistance bands, placed at the level of the knee, were introduced into the squat exercise to further increase the difficulty by applying additional force directed from the valgus direction thus recruiting greater stabilization activation of the hip abductors. The bands were placed laterally around the femoral condyles and remained taught as the participants squatted and completed calf and toe raises.

Participants performed stiff-leg deadlifts on the platform while holding a wooden stick to promote proper form. Initially, care was taken to promote spinal neutrality throughout the range of motion where spinal flexion would cue the cessation of the eccentric phase. By the conclusion of the training, all participants had performed the exercise with at minimum hip flexion of 45°, as flexibility allowed, while maintaining spinal neutrality. Stationary lunges were performed with the front foot on the platform and the back foot on the ground (Figure 1B). After lunging with one foot on the platform for the allotted time, they changed to the other foot and repeated the exercise to complete the set. Push-up holds were performed both with hands on the platform and then feet on the platform in an alternating fashion, per set (Figures 1C and 1D). Participants would assume a prone plank position with their arms extended. Upon initiation of vibration, the participant would flex the elbows to 90° and hold through the duration of the repetition with minimal shoulder abduction due to the width of the platform, while maintaining static body position throughout the core and lower extremity. Because of the challenging nature of this exercise, participants were allowed to decrease the frequency based on their personal abilities because emphasis was placed on technique in order to maintain a lower risk for injury. Push-up holds with the hands on the platform were frequently performed at a range 15–26 Hz, based on the strength of the participant. Bent-over rows were performed with identical form to the stiff-leg deadlift while moving a wooden stick through the desired range of motion with the upper extremity. Emphasis was placed more on the mechanics of the trunk rather than the movement of the arms. In week 9, jumps onto and off of the platform were introduced. When performing jumps participants started on the floor, jumped onto the vibrating platform and completed an exaggerated eccentric phase landing to half squat depth before holding this position statically for three seconds. To complete one repetition, they would then jump from the platform backwards off of the platform and onto the floor and repeat the 3 second hold.

### Bone Mineral Density

Bone mineral density (g/cm^2^) of the hip, lumbar spine, and whole body was assessed using dual energy x-ray absorptiometry (Hologic Explorer, Waltham, MA). The spine scans allow for analysis of the first four lumbar vertebrae (L_1_–L_4_) in the posterior-anterior view as well as three lumbar vertebrae (L_2_–L_4_) in the lateral view. All scans were performed and analyzed by the same lab technician. The coefficient of variation evaluating test-retest reliability of DXA scans, by this technician, at the Loyola Marymount University Human Performance Laboratory are 1.0% for BMD of the hip and spine.

### Statistics

Statistics were analyzed using SPSS software version 17.0 (Chicago, IL, USA). To be considered statistically significant, the alpha level was set at p ≤ 0.05. Standard descriptive statistics were performed on baseline data and are presented in Table 1. Pearson correlation coefficients were run to evaluate relationships between baseline anthropometric variables and BMD. Body height, body mass, BMI, nor lean body mass were related to BMD at the spine, however lean body mass was significantly related to total hip BMD (r= 0.503, p< 0.05) and whole body BMD (r= 0.517, p< 0.05). Assumptions for t-tests were confirmed by the Shapiro-Wilk test which revealed normal distributions and Levene tests confirmed homogeneity of variances for changes in BMD. A two-tailed t-test was then used to evaluate differences in the change in BMD at the hip, spine, and whole body between the control group and WBV group.

## Results

Participants in the WBV group adhered to 90% of the 12-week training program (range 74–100%) with only one female participant dropping out due to an unrelated injury, as stated previously in the methods. Changes in lean body mass over the 12 weeks were not significantly different between groups. The WBV group gained an average of 0.28±1.2 kg of lean mass while the control group lost an average of 0.49±1.8 kg. Lean body mass may explain 21–28% of the variation in BMD in premenopausal women and 18–73% of variation in BMD in athletic men (Lu et al., 2009; Rector et al., 2009). Because lean body mass has been reported to be related to BMD and because it was significantly different between groups at baseline, we evaluated its potential as a covariant in the statistical approach used here.

However, a two-tailed analysis of covariance (ANCOVA) revealed identical findings to the t-test and results did not vary whether or not lean body mass was controlled, therefore results of the t-test are presented.

Table 3 shows the average BMD values and standard deviations of WBV and control participants at baseline and at the 12-week follow-up for the posterior-anterior spine (L_1–4_), lateral spine (L_2–4_), total hip, and whole body. While changes in BMD at the hip and whole body were minimal and not significantly different between groups, there were differences in the change of BMD at the spine. The WBV group experienced significantly greater increases in BMD in thelateral view of the spine (p= 0.031), which is supported by a trend for greater increase in the posterior-anterior (PA) view (p= 0.079).

**Figure 2:**
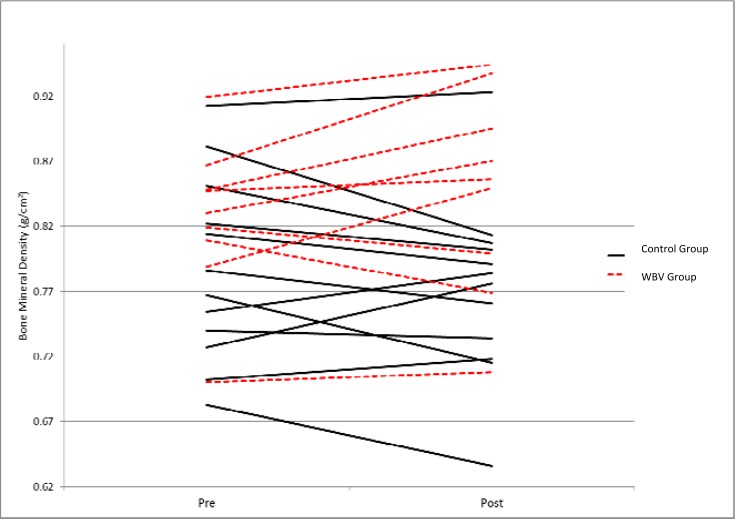
Change in lateral BMD for individuals in the control group (solid lines) and the WBV group (dotted lines) from baseline to post-training

**Figure 3:**
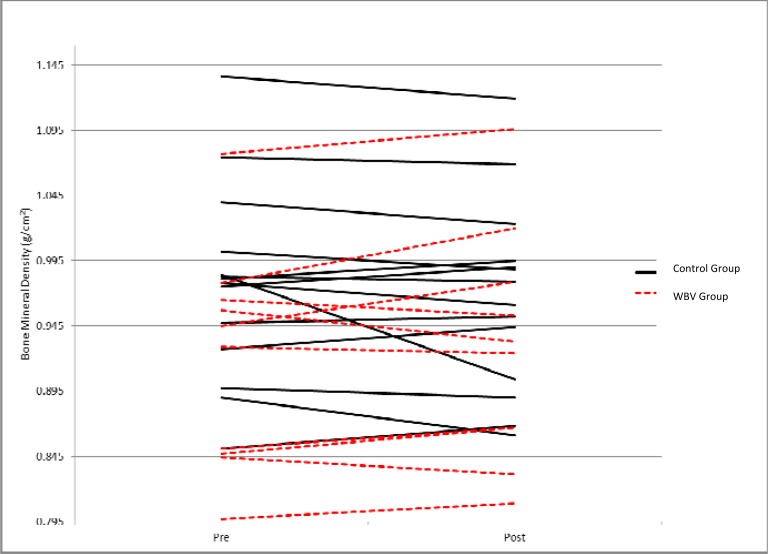
Change in BMD of the poster-anterior view of the spine for individuals in the control group (solid lines) and the WBV group (dotted lines) from baseline to post-training

Figure 2 displays bone mineral density at baseline and after the 12-week intervention at the lateral view of the spine. Figure 3 presents the baseline and post-training BMD values at the posterior-anterior spine. The WBV group (indicated by the dotted line) experienced an average increase in BMD of 2.7% in the lateral view and 1.0% in the posterior-anterior view of the spine. Meanwhile, the control group (solid line) showed an average decrease of 1.9% at the lateral spine and 0.9% at the posterior-anterior spine.

Results of the Block Food Frequency Questionnaire revealed a calcium intake of 1006±330 mg per day for the WBV group and 1009±298 mg per day for the control group (Table 1). Calcium intake measured at the 12-week follow-up via the LMU RAM was also similar between groups at 853±521 mg per day consumption for WBV group and 943±455 mg per day for the control group.

The menstrual history questionnaire revealed that 3 controls and 1 WBV participant were currently taking oral contraceptives. Of the 13 female controls, 10 reported eumenorrhea while three described their menstrual function as oligomenorrheic (more than 35 days between cycles). Three of the four female WBV participants experienced eumenorrhea while the remaining female reported to be amenorrheic.

## Discussion

The results from this investigation suggest that specific, dynamic exercise performed on a whole body vibration platform may be osteogenic. We report that a 12-week WBV training program performed 3 days per week improved BMD at the lateral and posterior-anterior view of the spine. Density changes may have been more significant at the lateral view due to its high content of trabecular bone, which is particularly responsive to changes in lifestyle patterns such as increasing weight-bearing activity. Conversely, the posterior-anterior view of the spine contains a higher proportion of cortical bone making up the spinous process; this type of bone is less responsive to rapid change when compared to trabecular bone. Report of lateral BMD is not yet commonplace in research literature and therefore its use in this investigation is a strength which adds to the body of work investigating bone health in response to exercise. The osteogenic success of this program after only 12 weeks of training is likely due to several factors such as the combination of vibration and dynamic exercise, the young age of the participants, high adherence of the supervised exercise program, lower baseline BMD at the spine, and an exercise program that progressively increased intensity.

While previous work has been done on the effects of WBV, little is known about how dynamic exercises performed on the vibration platform can increase bone health and assist in achieving optimal peak bone mass. This innovative training program combines WBV with exercises that have already been shown to elicit improvements in bone density. It is well known that bone responds to the physical deformation induced through weight-bearing activity by increasing density (Kohrt et al., 2004). The increase in BMD reported here may be due to the high level of strain caused by the dynamic exercises performed on the platform. Maddalozzo et al. (2007) reported that 12-months of squat and deadlift exercises improved BMD at the spine in postmenopausal women by 0.43%. While Maddalozzo et al. (2007) implemented a yearlong intervention (as compared to 12-weeks), this investigation may have found greater changes (2.7% advantage over controls) in a shorter period of time because the participants were younger (average age of 19), likely to be better trained, and additionally exposed to WBV during exercise. Increases in BMD at the spine were expected because the program specifically incorporated squat and deadlift exercises; movements that have been shown to improve BMD at the spine (Almstedt et al., 2011; Maddalozzo et al., 2007).

A high level of adherence strengthened the investigation as participants completed an average of 90% (range 74–100%) of the training program. The previously mentioned work by Gilsanz et al. (2006) found improvements in trabecular BMD at the spine and cortical bone area of the femur after female participants completed 12 months of WBV for 10 minutes a day. However, the findings of Gilsanz et al. (2006) were affected by a low compliance of 43%, most likely because the training was unmonitored and participants were asked to complete the vibration exposure on their own time, at home. A pilot investigation by Beck et al. (2006) installed platforms in the participants’ homes and experienced a mean compliance 60%. Even with compliance at 60%, the 12-month intervention by Beck et al. (2006) resulted in a 2% increase in BMD at the hip for women of about 38 years-of-age. The ability to detect improvements after only 12 weeks is likely influenced by the high adherence of this supervised exercise program.

The DXA bone scan provides a Z-score which reflects the comparison of a person’s bone mineral density to others of the same age, sex, and ethnicity. Z-scores are reported as the number of standard deviations above (positive values) or below (negative values) the average density of similar people. The International Society for Clinical Densitometry defines a Z-score of less than −2.0 as “below the expected range for age” (Bianchi et al., 2010). At baseline, participants exhibited normal BMD values at the hip, reflected by Z-scores close to the average (−0.02 for controls and −0.13 for WBV) and therefore likely had no major need for improvement in BMD at this bone site. Furthermore, at baseline, while still considered “normal”, participants had lower BMD at the spine, reflected by Z-scores of −0.46 for controls and −1.08 for WBV volunteers, which may better explain the success of the intervention, particularly at the spine. Moreover, when comparing this intervention design to the work of others, ours utilized dynamic exercises that progressively became more difficult or complex over the course of the program. Torvinen et al. (2003) incorporated some light squatting, jumping and slight knee flexion that increased in vibration frequency, however there were no evident improvements in the bone health of their young adult volunteers (ages 19–38 years) after 8 months of WBV training, 3–5 days per week. A possible cause for this success is the difficulty of the dynamic exercises, which progressively challenged participants.

Several researchers have investigated the potential for WBV to improve bone health for postmenopausal women. Beck and Norling (2010) reported that 8-months of WBV performed 2 times per week helped the postmenopausal volunteers to maintain BMD at the trochanter and spine while control counterparts experienced a 6% bone loss. In contrast, Bemben et al. (2010) reported no skeletal benefits to 8 months of WBV exposure (3 days per week) in combination with a traditional resistance training program. Improvements in BMD at the hip were observed by Gusi et al. (2006) who examined postmenopausal women also completing 8 months of WBV. Our investigation used a similar protocol to Gusi et al. (2006) including comparable total vibration time per session (approximately 360 seconds compared to our range of 165–540 seconds), use of the Galileo 2000 platform, which is the European partner of Vibraflex, and the similar high level of adherence (90%), however unlike Gusi et al. (2006) we did not find any statistically significant improvements in BMD at the hip, only the spine. A possible explanation for these findings at the spine but not at the hip is because participants were young, healthy and physically active at baseline, especially in activities that incorporated running. The WBV participants performed an average of 106.8+69.1 MET-hours per week of activity while the control group completed 65.4+49.4 MET-hours per week (Table 1). Despite this, physical activity outside of the intervention was not statistically different between groups, and statistically controlling for activity did not alter the findings. Because running is a weight-bearing exercise, it is often protective of bone health at the hip (Kohrt et al., 2004).

Intentional pieces of methodology decreased the opportunity for introducing error. All DXA scans were performed and analyzed by one, trained lab technician while all questionnaires were established as valid for this population (Block et al., 1990; Henry and Almstedt, 2009; Pereira et al., 1997). Calcium intake was measured because of its substantial potential influence on bone health. Calcium consumption measured via the Block 2005 Food Frequency Questionnaire at baseline or via the LMU RAM at follow-up was not statistically different between groups. According to the data from baseline, both groups were consuming more calcium (1006 ± 330 mg for WBV group vs. 1009 ± 298 mg for the control group, p=0.89) than the recommended daily allowance (RDA) of 1000 mg per day set by the Institute of Medicine for adults 19 years of age or older (Medicine, 2011). Dietary intake at follow-up indicated that both groups were continuing to consume similar amounts of calcium, although intake was slightly below the RDA at this time point (853 ± 521 mg for WBV group vs. 943 ± 455 mg for control group, p=0.66). Since calcium intake was similar between groups and consumption was above national levels for this age group, it is not likely that dietary intake of calcium influenced the bone results reported here (Alaimo et al., 1994).

It was unexpected to discover that control participants experienced a decrease in BMD during the 29 weeks between DXA scans (−1.9% at the lateral spine and −0.9% at the PA spine). These participants were asked to maintain their normal diet and exercise patterns and they demonstrated no significant changes in physical activity (MET-hours per week), dietary intake (mg per day of calcium), or body mass (kg). While the percent change could be due to measurement error associated with the DXA analysis, the combination of controls experiencing a decrease, while the WBV demonstrated an increase suggests the findings are due to the exercise intervention and not measurement error. With that said, a decrease in BMD for control participants warrants additional investigation into possible causes such as binge drinking or changes in the amount of bone-loading activity between high school and college years.

The objective of this investigation was to evaluate the osteogenic potential of WBV training with dynamic exercise at improving bone health in young individuals, thereby optimizing peak bone mass development. Results of this small investigation of a short duration are promising and provide justification for further evaluation, including correction of limitations to this pilot study such as the small sample size and 12-week intervention period. A longer training period may provide greater power to detect significant differences and would allow for periodic tracking of BMD changes. With so few participants, it was not possible to effectively evaluate bone improvements in men and women separately. Greater knowledge could be gained if this investigation were to be replicated with a larger sample size while investigating men and women separately.

A whole body vibration training program incorporating exercises such as squat, stiff-lead deadlift, stationary lunges, push-up hold, bent-over row, and jumps performed 3 days a week, for 12 weeks, improved spinal BMD in healthy, college-aged men and women. The program, which ranged in vibration frequency from 15–26 Hz, requiring 20–30 min per workout, elicited a positive change in vertebral bone mineral density. By increasing BMD in young adults, peak bone mass can be optimized and future risk for osteoporosis may be diminished. Further longitudinal investigation with a larger sample size is needed to assess the long-term effects of WBV training on the deterrence of osteoporosis.

## Figures and Tables

**Table 1:** Participant Characteristics at Baseline

	**WBV**	**Controls**
Sex	6 males, 4 females	1 male, 13 females
Age (years)	19.3 ± 1.3	19.8 ± 1.1
Body Height (cm)	174.5 ± 10.3	169.1 ± 6.5
Body Mass (kg)	60.8 ± 8.0	60.8 ± 8.8
BMI (kg/m^2^)	19.9 ± 1.1	21.3 ± 2.8
Body Fat (%)	17.3 ± 6.5	26.6 ± 4.2^*^
Lean Body Mass (kg)	47.3 ± 8.7^*^	41.5 ± 5.15
Calcium Intake (mg/day)	1006 ± 330	1009 ± 298
Physical Activity (MET-hrs/wk)	106.8 ± 69.1	65.4 ± 49.4

* significantly different between groups (p < 0.05) Values are presented in means ± standard deviations WBV= whole body vibration participants

**Table 2:** Whole Body Vibration Training Program

	**Standing**			**Squat**			**Deadlift**			**Lunge**			**Push Up Hold**			**Bent Over Row**			**Jumps**		
	Sec	Sets	Hz	Sec	Sets	Hz	Sec	Sets	Hz	Sec	Sets	Hz	Sec	Sets	Hz	Sec	Sets	Hz	Sec	Sets	Hz
Week 1																					
Day 1	30	3	15	30	2	15	30	2	15												
Day 2	30	3	20	30	2	15							15	1	15						
Day 3	30	3	25	30	2	20	30	2	15	30	2	15	15	2	15						
Week 2																					
Day 1	30	3	25	30	3	20	30	2	20	30	3	15	15	2	15						
Day 2	45	3	25	30	3	20	30	2	20				15	2	15						
Day 3	45	3	25	30	2	25	30	2	20	30	2	20	15	2	20						
Week 3																					
Day 1	45	3	25	60	3	25	30	3	25				15	3	25						
Day 2	60	3	25				30	3	25	60	3	25	15	3	25						
Day 3	60	3	25	60	4	25	45	2	25	60	4	25									
Week 4																					
Day 1	60	3	25	60	3	25	30	3	25							30	1	25			
Day 2	60	3	25				60	3	25	60	2	25	30	2	25	30	2	25			
Day 3	60	3	25	60	2	25	60	2	25	60	2	25	30	2	25						
Week 5																					
Day 1	60	3	25	60	4	25	60	3	25				30	3	25						
Day 2	60	3	25				60	3	25	60	3	25				30	3	25			
Day 3	60	3	25	60	3	25	60	3	25	60	2	25									
Week 6^*^																					
Day 1	60	2	26	60	4	25	60	4	25				30	4	25						
Day 2	60	2	26				60	4	25	60	3	25				60	3	25			
Day 3	60	2	26	60	3	25	60	2	25	60	1	25	30	2	25	60	1	25			
Week 7																					
Day 1	60	1	26	60	5	25	60	5	25				30	4	25						
Day 2	60	1	26				60	5	25	60	3	25				60	4	25			
Day 3	60	1	26	60	3	26	60	2	25	60	1	25	30	2	25	60	4	25			
Week 8																					
Day 1	60	1	26	60	5	26	60	5	25				30	4	25						
Day 2	60	1	26				60	5	25	60	3	25				60	3	25			
Day 3	60	1	26	60	3	26	60	2	25	60	1	25	30	2	25						
Week 9																					
Day 1	60	1	26	60	5	26	60	5	25				30	4	25						
Day 2	60	1	26				60	2	26	60	3	25				60	3	25	10	2	15
Day 3	60	1	26	60	3	26	60	2	26	60	1	25	30	4	25	60	2	25	10	3	15
Week 10																					
Day 1	60	1	26	60	5	26	60	5	26				30	4	25				10	2	20
Day 2	60	1	26				60	2	26	60	2	26				60	3	26	10	2	25
Day 3	60	1	26	60	3	26	60	2	26	60	1	26	30	4	25	60	2	26	10	2	25
Week 11^†^																					
Day 1	60	1	26	60	5	26	60	5	26				30	4	26				10	2	25
Day 2	60	1	26				60	2	26	60	2	26				60	3	26	10	2	25
Day 3	60	1	26	60	3	26	60	2	26	60	1	26	30	4	26	60	2	26	10	2	26
Week 12																					
Day 1	60	1	26	60	5	26	60	5	26				30	4	26				10	3	26
Day 2	60	1	26				60	2	26	60	2	26				60	3	26	10	3	26
Day 3	60	1	26	60	2	26	60	2	26	60	1	26	30	4	26	60	2	26	10	3	26

Sec = second; Rep = repetition; Hz = hertz

* incorporate calf and toe raises into the exercises;

† incorporate the use of bands around the knees

**Table 3:** Bone Mineral Density Results

**BMD Site (g/cm^2^)**	**WBV Group (n=10)**			**Control Group (n=14)**			
	Baseline	12-weeks	Change	Baseline	12-weeks	Change	p-value
PA Spine L_1–4_ BMD	0.919 ± 0.084	0.928 ± 0.089	0.009	0.976 ± 0.075	0.967 ± 0.074	−0.009	0.079
Lateral Spine L_2–4_ BMD^*^	0.825 ± 0.060	0.835 ± 0.082	0.022	0.787 ± 0.071	0.776 ± 0.068	−0.015	0.031^*^
Total Hip BMD	1.024 ± 0.114	1.015 ± 0.109	−0.008	0.981 ± 0.064	0.977 ± 0.071	−0.005	0.666
Whole Body BMD	1.123 ± 0.085	1.124 ± 0.086	0.001	1.119 ± 0.058	1.123 ± 0.060	0.005	0.618

BMD = bone mineral density in g/cm^2^; WBV = whole body vibration; PA= posterior anterior; L_1–4_= lumbar vertebrae 1–4; L_2–4_= lumbar vertebrae 2–4

* significantly different between groups (p < 0.05)
